# A Surgical Navigation System for Guiding Exact Cochleostomy Using Auditory Feedback: A Clinical Feasibility Study

**DOI:** 10.1155/2014/769659

**Published:** 2014-07-01

**Authors:** Byunghyun Cho, Nozomu Matsumoto, Shizuo Komune, Makoto Hashizume

**Affiliations:** ^1^Department of Advanced Medical Initiatives, Faculty of Medical Sciences, Kyushu University, 3-1-1 Maidashi, Higashi-ku, Fukuoka 812-8582, Japan; ^2^Department of Otorhinolaryngology, Graduate School of Medical Sciences, Kyushu University, 3-1-1 Maidashi, Higashi-ku, Fukuoka 812-8582, Japan

## Abstract

In cochlear implantation (CI), the insertion of the electrode array into the appropriate compartment of the cochlea, the scala tympani, is important for an optimal hearing outcome. The current surgical technique for CI depends primarily on the surgeon's skills and experience level to achieve the correct placement of the electrode array, and the surgeon needs to confirm that the exact placement is achieved prior to completing the procedure. Thus, a surgical navigation system can help the surgeon to access the scala tympani without injuring important organs in the complex structure of the temporal bone. However, the use of a surgical microscope has restricted the effectiveness of the surgical navigation because it has been difficult to deliver the navigational information to the surgeon from outside of the surgeon's visual attention. We herein present a clinical feasibility study of an auditory feedback function developed as a computer-surgeon interface that can guide the surgeon to the preset cochleostomy location. As a result, the surgeon could confirm that the drilling point was correct, while keeping his or her eyes focused on the microscope. The proposed interface reduced the common frustration that surgeons experience when using surgical navigation during otologic surgeries.

## 1. Introduction

Cochlear implantation (CI) has been becoming increasingly indicated for the treatment of sensorineural hearing loss [1]. The surgical technique for CI requires the insertion of an electrode into the cochlea to stimulate the auditory nerve, which consists of mastoidectomy and posterior tympanotomy to access the middle ear [2, 3]. This surgical technique involves drilling out the cortical bone and mastoid air cells to clearly identify anatomical landmarks in order to avoid injury to critical structures, for example, the facial nerve, the chorda tympani, and incus buttress, and the opening into the middle ear via the facial recess, which is a triangular region bounded by the facial nerve medially, the chorda tympani nerve laterally, and the fossa incudis superiorly. The round window (RW) of the cochlea and promontory are then identified through the posterior tympanotomy. The electrode array is inserted through the RW or a cochleostomy near the RW so that it is placed in the correct partition of the cochlea, which is called the scala tympani.

Although the surgical technique is well established, the risks and complications of the surgery, such as the injury of facial nerve, still remain [4, 5]. In addition, the risk increases in revision surgery or in patients with malformation of the ear, because these conditions make it more difficult for the surgeon to identify the anatomical landmarks of the temporal bone [6]. To reduce the risks during the procedure, surgical navigation systems have been increasingly used in otologic surgery [7–11]. Surgeons can perform procedures under image-guidance, in which the movement of the surgical drill can be simultaneously shown on the same screen as the preoperative CT or MRI. As the scala tympani of the cochlea is generally chosen as the point for inserting the electrode array, precise placement of the cochleostomy is required [12, 13]. A surgical navigation system may help the surgeon to accurately place the cochleostomy and to avoid drilling other critical structures within the same surgical area.

**Figure 1:**
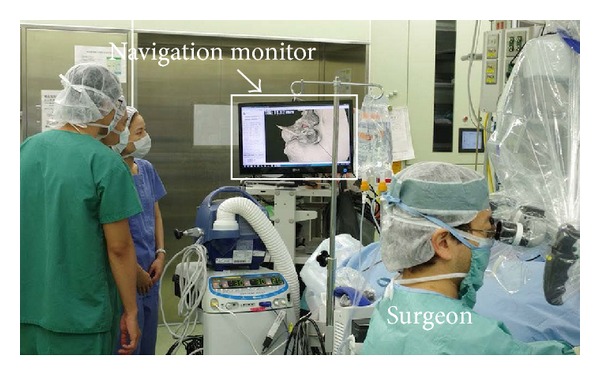
The typical situation in the operating room when using a conventional surgical navigation system. The surgeon cannot see the navigation monitor while drilling the bone.

However, there has been a common dilemma that otologists have experienced while using conventional navigation systems during otologic surgery, which is that the surgeon cannot continuously see the navigation monitor while drilling, because the surgeon must focus on the microscope view during the procedure (Figure 1). In order to see the navigation monitor, the surgeon's visual attention has to be moved from the operating field to the navigation monitor, which causes a temporary interruption of the surgical procedure. As such, the closer the surgical drill approaches to a critical anatomical region, the less the surgeon can access the navigation information that is shown outside the surgeon's visual field. For routine clinical use, a surgical navigation system should be able to provide the surgeon with accurate information without disturbing the surgery.

We have recently developed an auditory feedback system as a new interface between the surgeon and the surgical navigation computer. This new interface can deliver navigation information more effectively than a visual signal, without requiring the surgeon to move the visual focus [14, 15]. However, because auditory feedback interface can carry much less information compared to a computer screen, simple tones describing only the absolute distance to the target are not always sufficient for the surgeon to understand the current status of the surgery. The relative distance, for example, “is the drill closer to the round window than the oval window?” was often the information that the surgeon wanted to know.

We herein report our research on the development of a computer-surgeon interface that was developed to allow the navigation information in the computer to be more effectively delivered to the surgeon. Accurate placement of the cochleostomy using auditory feedback was developed to access the scala tympani as the target area. In addition to the simple feedback regarding the absolute distance to the target, a new algorithm to feed back the relative proximity to the target and the other nearby structures was developed. This new function improved the usefulness of the audible feedback system, because the surgeon could understand whether the drill was closer to the scala tympani or scala vestibuli, that is, the relative distance, in addition to the absolute distance. With this interface system, the surgeon could confirm the exact position of the scala tympani without needing to move the surgeon's visual attention. The proposed system, which can simultaneously provide information about the distance to multiple important organs within the surgical field, may be useful in other fields requiring image-guided surgery.

## 2. Materials and Methods

Institutional Review Board (IRB) approval was obtained from our institution. Each patient, or his/her guardian(s), provided written informed consent for the surgical procedures.

The 512 × 512 pixels dataset computed tomography (CT) images were acquired at a resolution of 0.12 mm/pixel and a 0.5 mm slice thickness. A free open source software program (3D Slicer, Version 3.6, Brigham Women's Hospital, Boston, MA, USA) was used as a software platform to display the information. An optical position sensor (Polaris, NDI, Waterloo, Canada) was used to track the movement of the surgical drill. The algorithm developed for the guidance system was incorporated into the tracking system software for the optical position sensor. A surgical drill with attached infrared markers was used for the surgical tool, and calibration files were prepared for drill burrs of various radii and lengths.

**Figure 2:**
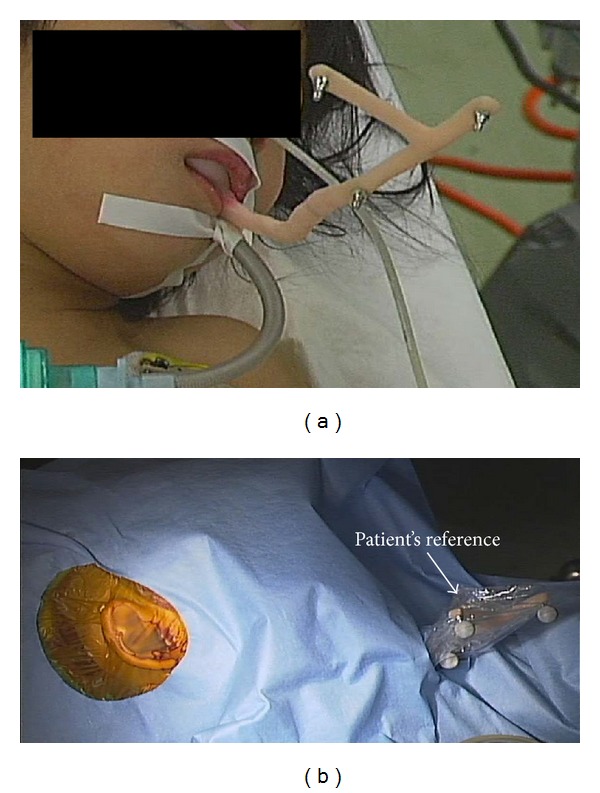
The minimally invasive mouth splint for the patient's reference (a) and its sterilization (b).

**Figure 3:**
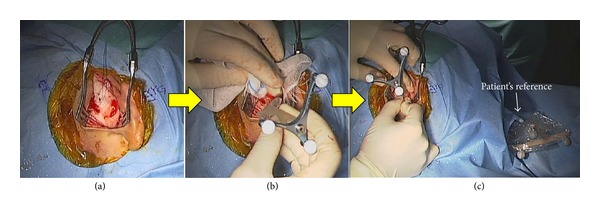
Image-to-patient registration using the preregistered STAMP method. The skin incision (a), the identification of the features by fitting the device on the surface of the temporal bone (b), and placing the template on the surface (c).

To compensate for and track the movement of the patient's head, a mouth splint that was fixed on the maxillary bone was used (Figure 2(a)). For sterilization, the mouth splint was first covered with a sterilized plastic drape and then sterilized infrared markers were mounted on the mouth splint (Figure 2(b)). Image-to-patient registration was accomplished by the preregistered STAMP method (Figure 3), which was developed in our laboratory for noninvasive image-guided temporal bone surgery [16, 17]. To ensure accurate registration, we saved the fiducial points deep in the temporal bone for intraoperative verification and updating, in addition to the surface points of the temporal bone for the STAMP method. This refinement improved the TRE in the deep region in the temporal bone by correcting the small error at the surface of the temporal bone (Figure 5).

### 2.1. Preparation for Warnings about the Absolute and Relative Proximity to the Target

We set the navigation software program to generate warning sounds when the drill tip entered a field within 5 mm from the target. Upon setting the facial nerve as a target, the frequency of the warning sound was set according to the absolute distance as reported previously [15], such that the warning tone was set at 300 Hz when the drill tip was closer than 5 mm, 600 Hz when the drill tip was closer than 3 mm, and 900 Hz when the drill tip was closer than 1 mm. Upon setting the scala tympani as a cochleostomy target, we set another target (scala vestibuli) that was adjacent to the scala tympani, where the drill should not enter. The navigation software program generated a warning sound when the drill tip approached within 5 mm of either of the targets. The tone was set according to the relative distance to the two targets; that is, the surgeon heard a high tone when the drill tip was closer to the scala tympani than to the scala vestibuli and a low tone when the drill tip was closer to the scala vestibuli than to the scala tympani.

### 2.2. System Configuration

**Figure 4:**
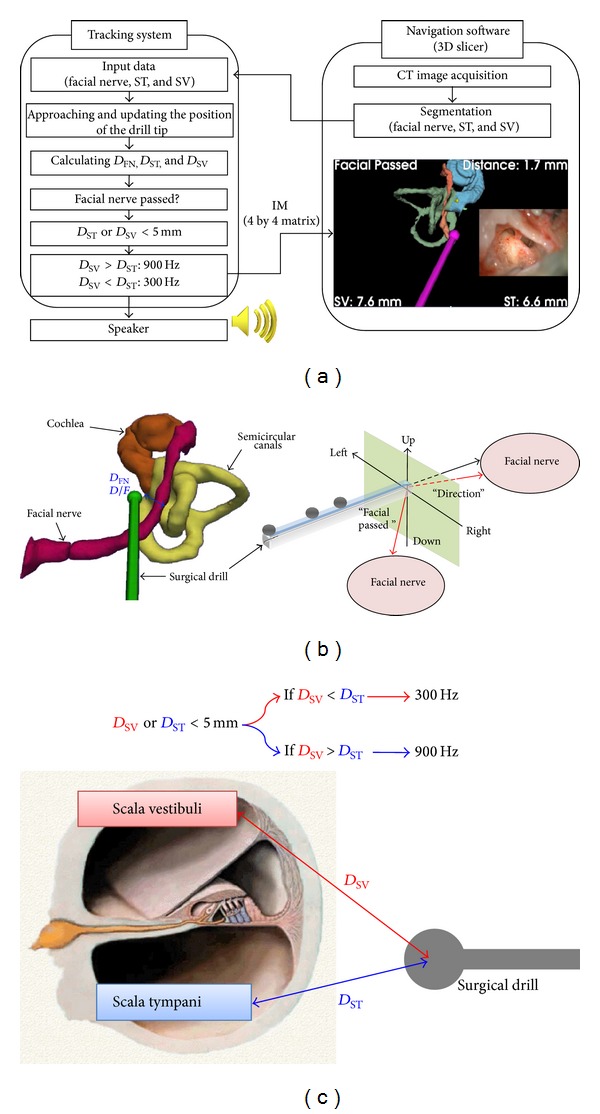
The configuration of the proposed surgical navigation system. (a) The flow chart of the proposed system, (b) monitoring the closest distance (*D*_FN_) between the facial nerve and the drill tip and its direction (*D*/*F*), and (c) the guidance for the scala tympani using auditory feedback with different frequency.

To use the proposed surgical navigation system, the following procedures were implemented as shown in Figure 4.

(1) Preoperatively, the surgeon manually performed image segmentation on preoperative CT images for small anatomical structures such as the facial nerve, scala tympani, scala vestibuli, cochlea, and semicircular canals. In terms of the scala tympani and scala vestibuli, the round window and oval window were used. Then, based on these segmentations, voxel data for the facial nerve, scala tympani, and scala vestibuli were computed and saved as text files.

(2) In the operating room, the saved voxel data were first input into the developed software program. When the drill tip approached the targets during drilling, the system continuously calculated the distances from the scala tympani (*D*_ST_) and scala vestibuli (*D*_SV_) to the drill tip and the closest distance between the surface of the facial nerve (*D*_FN_) and the drill tip. Then, the direction of the facial nerve relative to the drill tip (*D*/*F*) was computed to identify whether the drill had passed the facial nerve (Figure 4(b)). When the *D*_ST_ or *D*_SV_ was less than 5 mm, the system compared the distance between the *D*_ST_ and *D*_SV_. If *D*_SV_ > *D*_ST_, the system generated 900 Hz auditory feedback, while if *D*_SV_ < *D*_ST_, 300 Hz feedback was produced (Figure 4(c)).

(3) The computed data in a matrix form that included the *D*_ST_, *D*_SV_, *D*_FN_, *D*/*F*, and the position of the drill were transferred from the tracking system software program to the navigation software program. The matrix (IM) as image information had the following form:

(1) $$\begin{array}{c} \text{IM}=\left[\begin{array}{cc} R & t\\ \begin{array}{ccc} D_{\text{ST}} & D_{\text{SV}} & D_{\text{FN}} \end{array} & D/F \end{array}\right], \end{array}$$

where *R* is a 3 by 3 rotation matrix, *t* is a 3 by 1 translation vector for the drill tip, and *D*_ST_, *D*_SV_, and *D*_FN_ are the distances of the scala tympani (ST), scala vestibuli (SV), and facial nerve (FN) from the drill tip, respectively, and the *D*/*F* is the direction of the facial nerve relative to the drill tip. Each element in the transferred matrix is used to generate a display on the monitor of the navigation system.

## 3. Results

**Figure 5:**
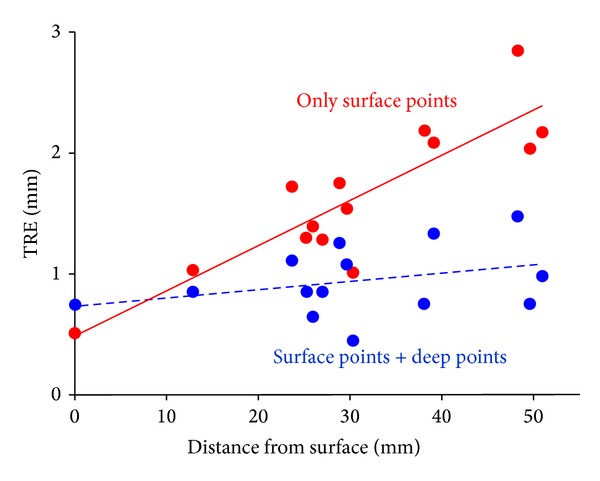
The measurement of the target registration error using the temporal bone replica.

To confirm the accuracy of the proposed system, we used a temporal bone replica to measure the target registration error (TRE) from the surface to the deep area in the temporal bone. Using virtual fiducial points on the surface and additional anatomical points in the deep region of the temporal bone for registration tended to be more accurate than using only the virtual fiducial points on the surface (Figure 5). During actual surgery, the surgeon confirmed the TREs using the round window as the target and the TREs in the whole area of the temporal bone were 0.8 ± 0.2 mm.

**Figure 6:**
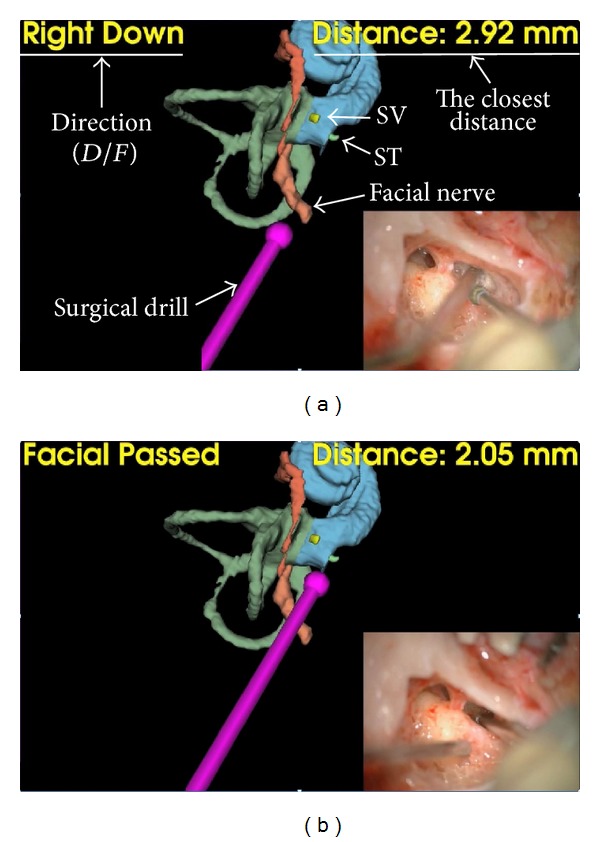
Monitoring the facial nerve (a) before and (b) after passing the facial nerve.

**Figure 7:**
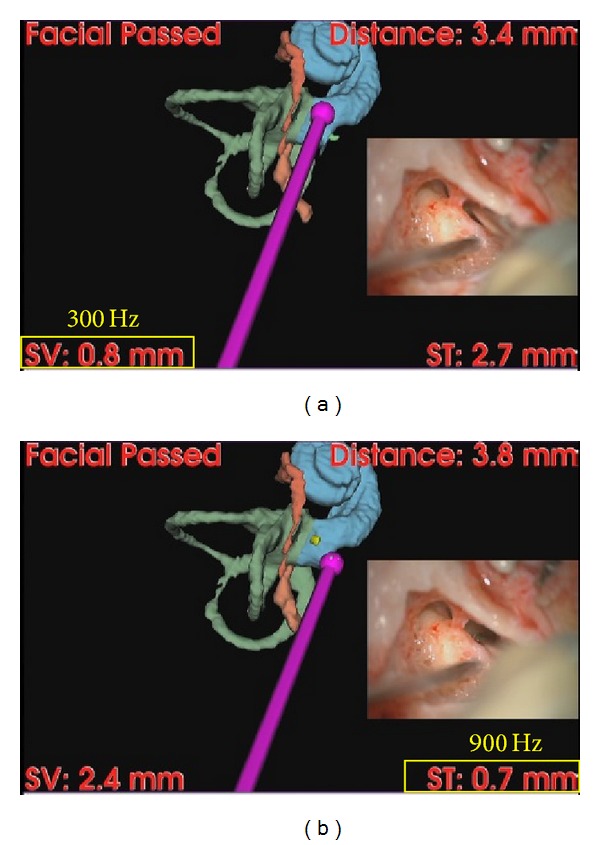
Confirmation of the exact placement of the cochleostomy using auditory feedback provided from the proposed system. (a) The scala vestibuli (300 Hz) and (b) the scala tympani (900 Hz).

Two patients successfully underwent CI surgery under image-guidance with the proposed interface. The system provided information about the closest distance from the drill tip to the surface of the facial nerve, along with the direction to the facial nerve. When the drill tip was shallower than the facial nerve, the system informed the surgeon about the distance and the direction (Figure 6(a)). Once the drill tip passed the facial nerve and, hence, the drill tip was deeper than the nerve, the system showed the note “facial passed” on the monitor to notify the surgeon that there was a lower risk of injuring the facial nerve (Figure 6(b)). Before cochleostomy, the surgeon confirmed the placement of the drill tip using the auditory feedback from the proposed system (Figure 7). When the surgeon intentionally pointed to a place near the stapes, which leads to the scala vestibuli, the system generated a low tone (300 Hz) indicating that the drill is relatively closer to the wrong scala of the cochlea (Figure 7(a)). On the other hand, the navigation system generated a high tone (900 Hz) when the surgeon placed the drill tip near the scala tympani, indicating that the drill tip is relatively closer to the correct scala (Figure 7(b)). Thus, the proposed system could lead the surgeon to the correct cochleostomy spot while letting the surgeon stay focused on the surgical microscope.

## 4. Discussion

Intraoperative facial nerve monitoring is widely used to identify the facial nerve while accessing the target during otologic surgery [18]. However, there are some limitations to its application. First, if an active stimulator is used, the stimulating electrode needs to be in physical contact on the bone near the facial nerve. Second, it is difficult to confirm the absolute distance from the facial nerve. Third, it is impossible to monitor the other organs that do not respond to electrical stimulation and generate electrical signals. Therefore, surgical navigation can be very useful to supplement the electrophysiological facial nerve monitoring. The present findings indicate that the auditory feedback system for the facial nerve is applicable for future routine clinical use because it could effectively inform the surgeon about the location of the drill tip during posterior tympanotomy, the procedure in which the facial nerve is closest to the drill and a point in time when the surgeon's visual attention cannot be moved from the operating field [19].

Technically speaking, surgical navigation with high accuracy is crucial for the reliability of the system. The preregistered STAMP method was an attractive registration method that could provide high accuracy in a noninvasive manner [15, 16]. The addition of deep points for intraoperative correction improved the TREs in the deep area in the temporal bone. The current registration error used in our method is close to the limit of the resolution of the optical tracking system. However, if a more accurate registration method becomes available in the future, or if some degree of invasiveness to improve the accuracy is justified, the proposed interface would show even greater usefulness.

There was some unavoidable time delay of the system when generating the auditory feedback [15]. To save the time used for the algorithm processing and data transfer, the calculations were performed by the tracking system software program for the optical position sensor, instead of by the navigation software program, and then the data were transferred to display information on the monitor. This bypassed some of the transfer processes within the computer. The resulting delay was 0.1-0.2 ms, and the surgeon did not recognize the delay during drilling.

In addition to the system that generates warning sounds, a simple algorithm that informs the surgeon about the relative distance to multiple structures was tested, with positive impressions reported by the surgeon. The proposed system could aid surgeons in making decisions where to place the cochleostomy, even though the distance between the scalae within the cochlea is very small [20]. We expect that a similar algorithm can be used in various cases when the surgical target is close to structures that must not be reached, for example, when a tumor is close to the jugular vein or the carotid artery.

## 5. Conclusions

We have demonstrated a surgical navigation system with a refined surgeon-computer interface for the placement of cochleostomy. The system informed the surgeon about the degree of the risk in advance using both visual signals and auditory feedback. The proposed system could lead the drill to the scala tympani without needing to avert the surgeon's visual attention from the surgical field. Therefore, the proposed interface showed a strong potential to enhance the use of an image-guidance system in routine CI.

## Supplementary Material

A navigation system that generating tones according to the relative distance to multiple targets. The surgeon is focusing on the microscopic image (inset) but is able to receive the relative proximity to the different scalae of the cochlea by hearing the different tones.
